# In silico identification and functional validation of linear cationic α-helical antimicrobial peptides in the ascidian *Ciona intestinalis*

**DOI:** 10.1038/s41598-020-69485-y

**Published:** 2020-07-28

**Authors:** Yukio Ohtsuka, Hidetoshi Inagaki

**Affiliations:** Biomedical Research Institute, National Institute of Advanced Industrial Science and Technology (AIST), 1-1-1 Higashi, Tsukuba, Ibaraki 305-8566 Japan

**Keywords:** Peptides, Bioinformatics

## Abstract

We developed a computing method to identify linear cationic α-helical antimicrobial peptides (LCAMPs) in the genome of *Ciona intestinalis* based on its structural and physicochemical features. Using this method, 22 candidates of *Ciona* LCAMPs, including well-known antimicrobial peptides, were identified from 21,975 non-redundant amino acid sequences in *Ciona* genome database, Ghost database. We also experimentally confirmed the antimicrobial activities of five LCAMP candidates, and three of them were found to be active in the presence of 500 mM NaCl, nearly equivalent to the salt concentration of seawater. Membrane topology prediction suggested that salt resistance of *Ciona* LCAMPs might be influenced by hydrophobic interactions between the peptide and membrane. Further, we applied our method to *Xenopus tropicalis* genome and found 11 LCAMP candidates. Thus, our method may serve as an effective and powerful tool for searching LCAMPs that are difficult to find using conventional homology-based methods.

## Introduction

Antimicrobial peptides are crucial and widespread effector molecules of the innate immune system and are a part of the first line of host defense against invading pathogens^1,2^. To date, thousands of antimicrobial peptides have been identified from bacteria to mammals and have been classified into several groups on basis of the structure and sequence features^3–5^. Of these, linear cationic α-helical antimicrobial peptides (LCAMPs), the genes of which encode short amphipathic peptides without cysteine residues, are the most abundant and widespread in organisms. LCAMPs are known for their broad-spectrum activities including the ability to rapidly kill or neutralize bacteria, fungi, viruses, parasites, and even cancer cells^6,7^. Therefore, they are considered promising lead candidates for the development of new peptide antibiotics^8^. There is a need for novel antibiotics due to the growing problem of microbial resistance to conventional antibiotics. LCAMPs might reduce the risk of emergence of antibiotic-resistant bacteria. In vitro development-resistance studies showed that pexiganan, an LCAMP, possesses a low potential for induction of bacterial resistance^9^.

Marine organisms are considered the richest source of novel antimicrobial peptides. They live in close proximity with pathogenic microbes. The estimated density of bacteria in seawater is approximately 10^6^ bacteria/mL^10^. In order to survive in this environment, they need to have a robust and effective immune system. Marine organisms are also exposed to extreme conditions, such as low temperature, high salinity and elevated pressure. The diversity of the marine environment has provided novel and unique sources of potential antimicrobial peptides^11^. In fact, marine antimicrobial peptides were found to be structurally different from their analogs in terrestrial species^12^. However, relatively limited numbers of antimicrobial peptides have been isolated from marine organisms, because antimicrobial peptides are a rapidly evolving group and display low sequence homology between orthologous antimicrobial peptides. Leoni et al*.* successfully identified a novel LCAMP family in marine mussels by bioinformatics analyses of available genomic and transcriptomic data^13^. To effectively and accurately identify novel antimicrobial peptides, there is a need to develop additional bioinformatics tools to survey protein sequence databases without use of sequence homology.

The ascidian *Ciona intestinalis* type A (hereinafter referred to as *C. intestinalis*) is a well-characterized marine invertebrate. Its genome has been sequenced^14^ and a large quantity of information about its transcriptome has been collected by EST and RNA-seq analyses^15,16^. It has been concluded that among invertebrates, ascidians are the closest phylogenetic relatives to vertebrates. Moreover, genome-wide sequence analysis revealed that they have a set of genes for innate immune system, such as complement factors and Toll-like receptors, but lack an adaptive immune system that is present in higher vertebrates^17,18^. These findings suggest that innate immune system for host defense is highly evolved in ascidians.

Previously, two salt-resistant LCAMPs, Ci-MAM-A and Ci-PAP-A, were identified from *C. intestinalis* hemocyte EST database by searching signal peptide and cationic helical region^19,20^. In this study, to identify further novel LCAMPs in *C. intestinalis*, we developed an in silico screening method based on further criteria such as the size, amphipathicity and aggregation propensity. Consequently, we computationally predicted 22 potential LCAMP candidates in *Ciona* genome and experimentally confirmed five novel salt-resistant LCAMPs with broad-spectrum antimicrobial activity. This strategy was also successfully applied to *Xenopus tropicalis* genome, suggesting that our method could be applicable to the in silico screening of any genome.

## Results

### Genome-wide search for discovery of novel *Ciona* LCAMPs

We developed a computing method for the detection of LCAMPs in the *Ciona* genome based on the structural and physicochemical features of known LCAMP precursors: short length, secretory peptide, and cationic amphipathic α-helix of mature peptide. The *Ciona* genomic database called Ghost database, includes 21,975 non-redundant complete amino acid sequences, from which 1,949 sequences with 50–100 residues in length were extracted.

Firstly, to find LCAMP precursors from 1,949 sequences, we predicted signal peptides using SignalP 4.1 server. In total, 252 sequences were predicted to be secretory peptides, and the sequences corresponding to the signal peptides were eliminated from them. Furthermore, the secretion of their peptides into extracellular space was confirmed using TMHMM server, and then, eight sequences were eliminated as transmembrane proteins. Next, sequences containing cysteine residues were eliminated from the 244 subsequences without signal peptides, and cationic amphipathic α-helical structure was predicted using HeliQuest server. HeliQuest screening was performed for 165 subsequences without cysteine residues and 80 putative cationic amphipathic helical segments were identified. The secondary structure of HeliQuest-identified cationic amphipathic segments was further confirmed. Using PROTEUS server, it was found that 44 segments had the potential to form α-helical structures. Subsequently, the aggregation propensity of cationic amphipathic α-helical segments was predicted using AGGRESCAN, in which normalized average of aggregation propensity (Na^4^vSS) of the proteins was evaluated. Forty-four cationic amphipathic α-helical segments had Na^4^vSS values within a broad range of − 65.6 to 14.8. A previous study reported that antimicrobial peptides have Na^4^vSS values within the range of − 40 to 60^21^. Therefore, we investigated whether cationic amphipathic α-helical segments with Na^4^vSS value less than − 30 have antimicrobial activity. Four selected segments, KH.C1.365, KH.L63.9, KH.S1007.2, and KH.S930.2 with Na^4^vSS values of − 32.9, − 65.6, − 48.1 and − 44.4, respectively, exhibited no or weak antimicrobial activities against *Escherichia coli* (Supplementary Fig. S1). Consequently, as potential candidates for *Ciona* LCAMPs, we identified 24 segments with Na^4^vSS value between − 30 and 30. Finally, the prediction of subcellular localization was performed to eliminate cellular components from LCAMP candidates. Using DeepLoc-1.0 server, 22 genes were identified as LCAMP candidates (Table 1).

**Table 1:** The list of LCAMP candidates predicted from *Ciona* genome database.

Gene ID	Cationic amphipathic helix sequence	Anionic helix sequence	Putative protein product
KH.C1.100	WSSLGRSLLRLTHALKPLA	FAPEIEDELEASEMDRIMQQMADEKQ	Ci-MAM-A
KH.C1.1006	WTVTRYWNQKLANLLAGK		Uncharacterized protein
KH.C1.1192	DGWVRTGLAVARLVVGRRRRRWNEANGL	RWNEANGLEKLSSDAEETLSAAEMEEVMQKIMDHQ	Ci-MAM-D^a^
KH.C1.1268	DWVRTAIGVAGLVLGRRRRGGWNQANGLKKFSSDA	LKKFSSDAEETLSAAEMEEVMQKIMDHQ	Ci-MAM-E^a^
KH.C1.453	ALRSAVRTVARVGRAVLPHV	QQPTSQADMLEDALEAQAIEALMQE	Ci-PAP-A
KH.C1.640	WLSRRRSSLFYWRRRRYDQ	RRYDQQSAKDMNEEDEPAETEAISDMLAKEDMPE	Uncharacterized protein
KH.C10.625	VFPSRRYGSLWSSFRRRIIRIHPQP		Uncharacterized protein
KH.C14.152	FRGLVRAGGKLVKEVLPSI	PWTKWNEQERMALADEIDAELMDLLDQ	Ci-META4
KH.C14.235	FRGLVRAGGKLVKEVLPSI	PWTKWNEQERMALADEIDAELMDLLEQ	Ci-META4
KH.C4.429	RLPQTVKNSLKHDQVIWKLFVKGV		Uncharacterized protein
KH.C5.577	EHVFFSRRRRWTRWNQKVVVEDI	WNQKVVVEDILEAMEQSDMLH	Uncharacterized protein
KH.C7.94	FSRRRFDFSRRRIYVARRRSLAFAHRRRFGDTA	NNPIKTDSDETSYIHSDQADDELMQMAE	Uncharacterized protein
KH.L136.4	WWLSGRRRVGRRRRRIIAY		Uncharacterized protein
KH.L14.3	YNARDLAKRNVGVSGQRVVSI	LGDTADINEYLSRLLDYES	Uncharacterized protein
KH.S1531.4	INKKFRWHGKRKWWLRFVKQYSGNENIQ		Uncharacterized protein
KH.S2775.1	KGRSLKKIRQFWRKFYKPFR	NENIQRNFLNMERESIEEMMADEIFRNF	Uncharacterized protein
KH.S655.3	RRRRIAGKIGGGVAKTAAEL	TAAELAAEQALESSTGGGSWS	Ci-META6
		WNQQRKMENAMNDEMDAELLLNLLKE	
KH.S775.4	SLLRVETKAILGTLALRRRTWNENKASQQITP	ITPEMEEKLDAEMEKLMQQLAEDQ	similar to Ci-MAM-A
KH.S775.5	SLAAGFKLLFKSWVHRRRTWNEKTTSD	NEKTTSDQFTAENDKPFDFAEMDGLLNNLETENQ	Ci-MAM-C^a^
KH.S908.1	KGGKFLNFLKKAAKVGAKVGMAAL	MAALGDEGEIEAFERLDTETQHAILAEALEN	Uncharacterized protein
KH.S921.1	TWPKNYWRKVWSKKNWRKFVKKFKHWNQGQNVED	KFKHWNQGQNVEDMDLEDMQLLWE	Uncharacterized protein
KH.S921.3	QAGVFDRKFWTRKHWSQVGKGLKRWNQKQNVENM	RWNQKQNVENMDLDDEIQYYE	Uncharacterized protein

^a^Nomenclature of LCAMPs by Fedders et al*.*^19^.

**Figure 1:**
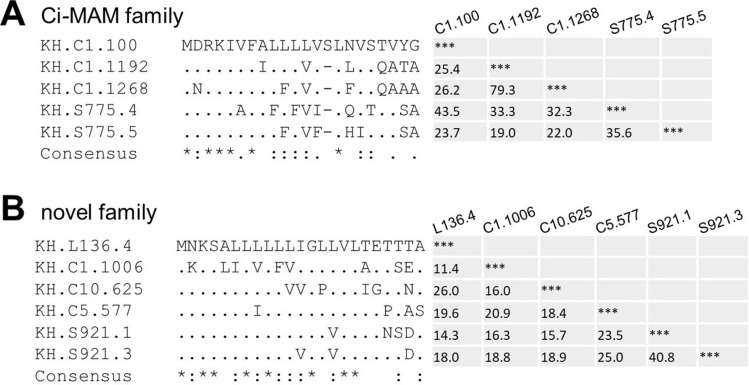
Multiple sequence alignment of the signal peptide and identity matrix in LCAMP families. Sequences of the predicted signal peptides in Ci-MAM (**A**) and novel (**B**) families were aligned using ClustalW. Percentage identity values for pairwise comparisons of the sequences without signal peptides of these family genes are shown.

*Ciona* LCAMP candidates identified by our screening method include known LCAMPs such as Ci-MAM-family genes^20^ and a number of uncharacterized proteins. In addition to *Ciona* genome, we predicted LCAMPs in *X. tropicalis* genome using our screening method. Eleven LCAMP candidates containing seven known LCAMPs^22^ were selected from 26,132 non-redundant protein sequences in the *X. tropicalis* genome (Supplementary Table S1), which suggests that our screening method is able to support the identification of LCAMPs from various genomes. LCAMP precursors are known to have a highly conserved signal peptide^23^. Two conserved signal peptides were found in *Ciona* LCAMP candidates identified by our screening method (Fig. 1). In five members of Ci-MAM family, signal peptides composed of 21–22 amino acid residues were well-conserved (80% majority-rule consensus sequence: MDRKIVFALXLVXXLXVSXXXA). In addition, another conserved signal peptide composed of 23 amino acid residues (80% majority-rule consensus sequence: MNKSALLXLLXXGLXVLXEXXXA) was identified in six LCAMP candidates, suggesting that they belong to a novel LCAMP family. Previous studies showed that *Ciona* LCAMP precursors possess an anionic helical region in addition to a cationic amphipathic region^19,20^. Using HeliQuest server, anionic helical sequences were found at the C-terminus of 17*Ciona* LCAMP precursor candidates (Table 1), indicating that most *Ciona* LCAMP precursors are composed of a signal peptide, a cationic amphipathic region, and an anionic helical region in that order.

### Antimicrobial activity of *Ciona* LCAMPs

Uncharacterized proteins in Table 1 are promising candidates of novel LCAMPs. To identify novel LCAMPs in *Ciona*, we tested antimicrobial activity in five LCAMP candidates, KH.C1.640, KH.C7.94, KH.S1531.4, KH.S908.1, and KH.S921.1. All synthesized peptides exhibited a broad-spectrum of antimicrobial activity (Table 2). They had potent antimicrobial activity against two anaerobic bacteria *E. coli* and *Staphylococcus aureus*. KH.S908.1 and KH.S921.1 peptides also showed potent antimicrobial activity against aerobic bacteria *Pseudomonas aeruginosa*. Furthermore, antifungal activity against *Saccharomyces cerevisiae* was observed in KH.C1.640, KH.C7.94, KH.S1531.4, and KH.S908.1 peptides. These results show that our screening method is capable of identifying novel LCAMPs.

**Table 2:** Antimicrobial properties of *Ciona* LCAMPs.

Peptide	MIC (μM)			
	*E. coli *(NBRC 14237)	*S. aureus *(NBRC 12732)	*S. cerevisiae *(NBRC 10217)	*P. aeruginosa *(NBRC 12582)
KH.C1.640	< 3.125	< 3.125	12.5	Negative
KH.C7.94	6.25	6.25	6.25	Negative
KH.S1531.4	< 3.125	< 3.125	6.25	Negative
KH.S908.1	< 3.125	< 3.125	6.25	6.25
KH.S921.1	< 3.125	< 3.125	Negative	< 3.125

**Figure 2:**
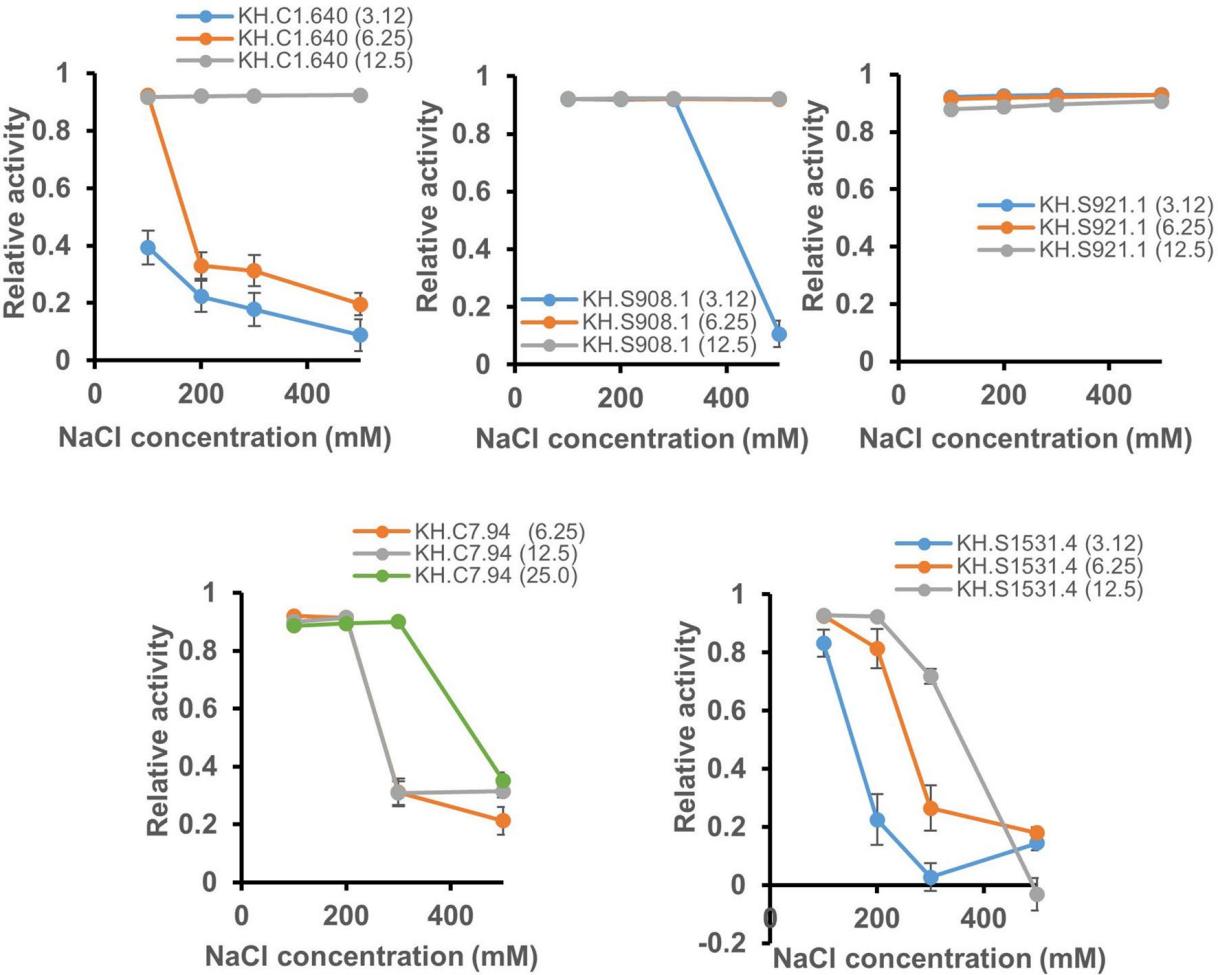
Effect of salt on the antimicrobial activity of *Ciona* LCAMPs. Antimicrobial activity of KH.C1.640, KH.C7.94, KH.S1531.4, KH.S908.1, and KH.S921.1 peptides against *E. coli* was assessed in the presence of 100, 200, 300, and 500 mM NaCl. Peptide concentrations were 3.12, 6.25, and 12.5 μM. Error bars show the standard deviation (*n* = 3) from a single experiment.

Ci-PAP-A (KH.C1.453) and Ci-MAM-A (KH.C1.100) are known to exhibit antimicrobial activity in the presence of 100 and 450 mM NaCl, respectively^19,20^. We examined the effects of five novel *Ciona* LCAMPs in the presence of NaCl on antimicrobial activity against *E. coli*. Reflective of the habitat of *C. intestinalis*, salt resistance was found in all peptides at the concentration of 12.5 μM (Fig. 2). In particular, KH.C1.640, KH.S908.1, and KH.S921.1 peptides retained potent antimicrobial activity in the presence of up to 500 mM NaCl. Previous studies suggest that helical stability, hydrophobicity and amphipathicity in LCAMPs are important for salt resistance^24–27^. However, each property of *Ciona* LCAMPs cannot explain salt resistance per se, as shown in Table 3. Therefore, we predicted the membrane interaction property of *Ciona* LCAMPs using AmphipaSeek server, which predicts that a protein or peptide can be monotopic and anchored via an amphipathic helix inserted parallel to the membrane interface^28^. The prediction showed that a membrane anchor region was found in higher salt resistant LCAMPs (Ci-MAM-A, KH.C1.640, KH.S908.1, and KH.S921.1) but not in low-salt resistant LCAMPs (Ci-PAP-A and KH.C7.94; Table 3). This suggests that in-plane membrane anchoring might be responsible for salt resistance.

**Table 3:** Physicochemical properties of *Ciona* LCAMPs.

Gene ID	Antimicrobial peptide sequence	Hydrophobicity	Hydrophobic moment	Net charge
KH.C1.640	W**WLSRR**RSSLFYWR	0.624	0.170	+ 4
KH.C7.94	SRRRFDFSRRRIYVARRRSLAFAHRRR	− 0.033	0.118	+ 11
KH.S1531.4	**TINKKFRWHGKRKWWLRFVKQ**	0.316	0.151	+ 8
KH.S908.1	**KGGKFLNFLKKAAK**VGAKVGMAALG	0.294	0.319	+ 6
KH.S921.1	**KLKTWPKNYWRKVWSKKNWRKFVKKFKHW**	0.272	0.141	+ 12
KH.C1.100 (Ci-MAM-A)	WR**SLGRTLLRL**SHALKPLARRSGW	0.428	0.380	+ 6
KH.C1.453 (Ci-PAP-A)	ALRSAVRTVARVGRAVLPHVAI	0.449	0.350	+ 4

The membrane anchor segments predicted by AmphipaSeeK are highlighted in bold.

The hydrophobicity, hydrophobic moment, and net charge values calculated by HeliQuest.

### LCAMP gene expression in *Ciona* hemocytes

**Figure 3:**
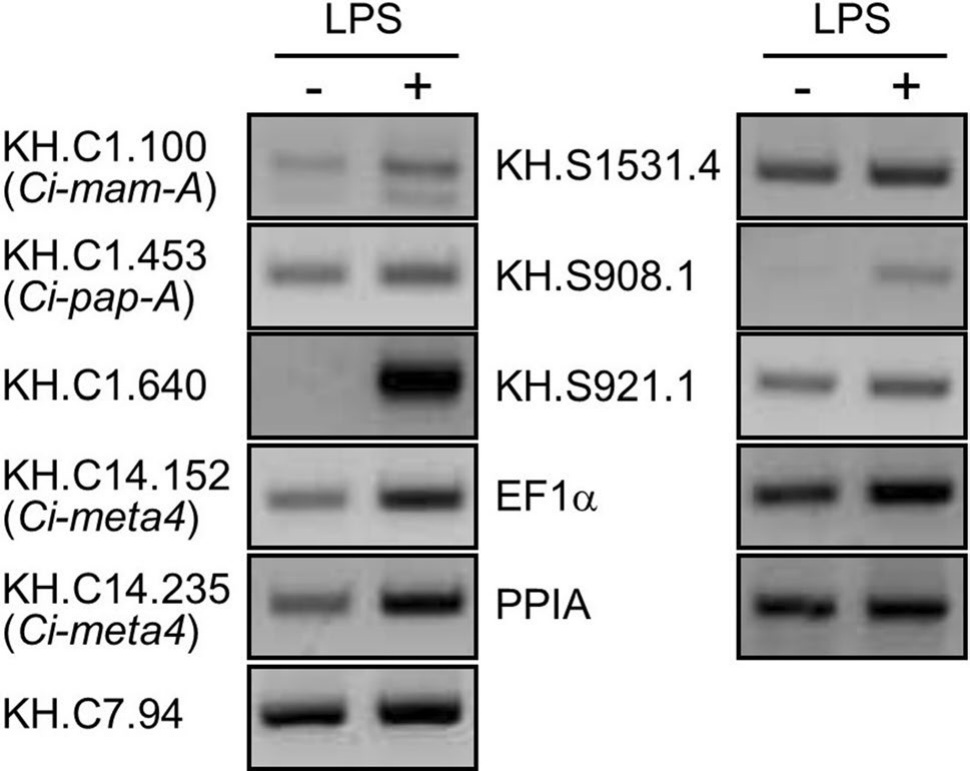
LCAMP mRNA expression in *Ciona* hemocytes stimulated with LPS. The expression of LCAMP genes in *Ciona* hemocytes treated (+) or untreated (−) with 0.1 mg/mL LPS for 1 h was analyzed by RT-PCR. EF1α and PPIA were used as internal controls.

To investigate the expression of *Ciona* LCAMPs in response to microbial infections, we performed RT-PCR analysis on *Ciona* hemocytes stimulated with bacterial lipopolysaccharide (LPS). First, the expression of *Ci-mam-A* (KH.C1.100), known as an inducible antimicrobial peptide^20^, was examined using our culture system. As a previous study, *Ci-mam-A* expression was induced by LPS stimulation (Fig. 3). The expression of eight LCAMP genes in LPS-treated hemocytes was also further assessed by RT-PCR. *Ci-meta4* and two novel LCAMP genes (KH.C1.640 and KH.S908.1) were transcriptionally upregulated by LPS, whereas there were no changes in four LCAMP genes containing *Ci-pap-A* (Fig. 3). NF-κB is activated in cells challenged with LPS and other inflammatory stimuli and is involved in the transcriptional activation of responsive genes^29^. To confirm the involvement of *Ciona* LCAMPs in the immune response, we predicted NF-κB binding sites in the upstream regions of each gene. Using Match-1.0 public, putative NF-κB binding sites were identified in the upstream regions of LPS-inducible LCAMP genes, but not in the upstream regions of KH.C7.94 and KH. S1531.4 genes (Table 4). These suggests that four inducible LCAMPs (Ci-MAM-A, Ci-META4, KH.C1.640, and KH.S908.1) may play a role in the innate immune response.

**Table 4:** Putative NF-κB binding sites in the upstream region of *Ciona* LCAMP genes.

Gene ID	Positions from ATG start codon
KH.C1.100	− 99/− 108, − 480/− 493, − 1,291/− 1,300
KH.C1.453	− 523/− 532, − 545/− 554, − 1,121/− 1,131, − 1,210/− 1,221, − 1,235/− 1,248, − 1,635/− 1,648
KH.C1.640	− 286/− 299
KH.C14.152	− 37/− 46, − 243/− 253, − 1,449/− 1,458
KH.C14.235	− 1,446/− 1,455
KH.C7.94	N.D.
KH.S1531.4	N.D.
KH.S908.1	− 692/− 701, − 747/− 756
KH.S921.1	− 124/− 133, − 811/− 824, − 1,290/− 1,299

*N.D. not detected.

## Discussion

In this study, we applied a computational approach to identify novel LCAMPs in *C. intestinalis* and identified 22 potential LCAMP candidates using their known physicochemical characteristics and subcellular localization predictions. Furthermore, we experimentally validated the broad-spectrum antimicrobial activity of five selected LCAMPs. Two of them were induced with LPS and putative NF-κB binding sites were identified in the upstream region of their genes, suggesting that they may play a role in the innate immune response. However, our data do not rule out the involvement of the other three genes in the immune response, as they may be regulated by uncharacterized innate immune signaling pathways.

In order to explore the proteins and peptides with similar function among different species, homology search tools such as BLAST have generally been used. Several defensin homologs were identified by BLAST search in plant and vertebrate genomes^30–32^. In these studies, some defensins that could be missed by BLAST search were further identified by a hidden Markov model based on a conserved cysteine-rich defensin motif. In contrast to defensin with three unique cysteine frameworks, it is difficult to find LCAMPs among different species using commonly used homology search tools due to poor sequence conservation of LCAMPs, especially in the cationic amphipathic α-helical region^33^. Therefore, computational approaches without sequence homology information have been designed to discover novel LCAMPs from sequence databases. In marine mussels, novel antimicrobial peptides, myticalins, were identified in the genomes and transcriptomes using bioinformatics analyses^13^. In *Ciona*, two LCAMP families, Ci-MAM and Ci-PAP, were identified from a hemocyte EST database based on two structural criteria of LCAMPs, signal peptide, and cationic α-helical region^19,20^. We developed an in silico method for screening LCAMPs based on seven criteria, size, cationicity, hydrophobicity, amphipathicity, helicity, aggregation propensity, and subcellular location. Thus, we identified peptides that potentially have antimicrobial activity based on the structural and physicochemical features and eliminated non-antimicrobial peptides which cannot come into contact with microbes using subcellular localization prediction. In the prediction of these properties, we used web-based tools that can handle a large number of sequences simultaneously (see “Materials and methods”). This enables screening of LCAMPs in genome databases. Consequently, we identified novel LCAMPs containing KH.S921.1 from *C. intestinalis* genome database using our in silico method. In addition to *C. intestinalis* genome*,* we applied this method to *X. tropicalis* and succeeded in identifying the cationic amphipathic region including known LCAMPs. Our newly developed method is effective in screening LCAMPs in the genomes of various species. However, our in silico method does not completely exclude false positives. Experimental validation is necessary to validate novel LCAMPs in other organisms using our screening method.

A number of LCAMP precursors are known to possess a signal peptide, cationic amphipathic region, and anionic proregion^34,35^. In *C. intestinalis*, almost all candidates shared a typical primary structure composed of a signal peptide, a cationic amphipathic region, and an anionic helical proregion in that order. The signal peptide is highly conserved within the species, and this conservation tendency increases within the individual LCAMP families. For instance, the signal peptides of Ci-MAM family genes are well-conserved^20^ (Fig. 1). We identified a novel conserved signal peptide composed of 23 amino acid residues, indicating that six LCAMP precursors containing KH.S921.1 with their conserved signal peptide form a novel LCAMP family. In this novel LCAMP family, the anionic proregion is short or absent, whereas in the Ci-MAM family, it is relatively conserved. LCAMP precursors require proteolytic processing in which anionic proregions are removed by proteolytic cleavage to attain their active forms. In frog, LCAMP precursors are processed at the RXXR-, KK-, or RR-motifs corresponding to common cleavage sites in proregions^22,23^. However, in *Ciona* LCAMP candidates, acidic amino acid residues, E and D, were found around the boundary of putative cationic active region and proregion, but their basic processing motifs were not. In ascidian, LCAMP precursors may be processed at the N-terminal of the acidic amino acid residue. Previous studies showed that ascidian LCAMPs, Styelins and Clavanins, are cleaved at the N-terminal of D and DD, respectively^36,37^.

Herein, we demonstrated salt resistance of *Ciona* LCAMPs. Many antimicrobial peptides lose their activities under physiological salt concentration, although they exhibit significant in vitro activity against bacteria in the presence of low salt concentration. Considering the therapeutic application, the salt-dependent inactivation of antimicrobial peptides is a major obstacle^38^. Marine organisms, including *C. intestinalis,* are considered a good resource of salt-resistant antimicrobial peptides*.* So far, several salt-resistant antimicrobial peptides have been identified in marine organisms^39^. We demonstrated that three synthetic peptides derived from KH.C1.640, KH.S908.1, and KH.S921.1 retained potent antimicrobial activity in the presence of up to 500 mM NaCl. Additionally, AmphipaSeek prediction strongly suggested these peptides appear to insert parallel to membrane interface. We propose that salt resistance is influenced by hydrophobic interaction between the peptide and membrane. The action mechanism of LCAMPs, such as dermaseptins and cecropins, is explained by “carpet model”^40^. According to this model, the peptides first align parallel to the phospholipid bilayer membrane, remaining in contact with the lipid head groups and effectively coating the surrounding area. When a threshold concentration of the peptide is reached, the peptides self-assemble within the membrane plane and this reorientation leads to a local disturbance in membrane stability, causing the formation of large cracks, leakage of cytoplasmic components, disruption of the membrane potential, and ultimately disintegration of the membrane. High-salt concentration tends to disturb the first step of the action mechanism, especially the electrostatic interaction between cationic peptides and negatively charged bacterial membranes. It seems likely that salt-resistant LCAMPs could be bound to the membrane surface by the hydrophobic interaction, even if the electrostatic interaction was disturbed under high-salt conditions.

In this study, we demonstrated the effectiveness of a combination of computational and experimental approaches used to identify putative and novel LCAMPs from ascidian and frog genomes. LCAMP is a promising candidate for a new class of antibiotics, with a broad spectrum of antimicrobial activity, ease of synthesis, and a novel mechanism of action against microbes. Our in silico screening method will allow us to discover novel LCAMPs from genetic resources and shed light on the relationship between the structure and function of LCAMPs.

## Materials and methods

### Genome-wide in silico screening for antimicrobial peptides

The amino acid sequences used for discovery of novel LCAMPs were retrieved from two genome databases, *Ciona intestinalis* type A (recently called *Ciona robusta*) sequences from Ghost database (https://ghost.zool.kyoto-u.ac.jp/cgi-bin/gb2/gbrowse/kh/)^41^ and *Xenopus tropicalis* sequences from Xenbase (https://www.xenbase.org/entry/)^42^. LCAMP prediction was performed for sequences with 50–100 residues in length. LCAMP candidates were selected based on signal peptide sequence, hydrophobicity, hydrophobic moment, net charge, α-helix structure, and aggregation propensity. The signal peptide searches and cleavage site predictions were carried out using SignalP 4.1 server (https://www.cbs.dtu.dk/services/SignalP-4.1/)^43^. Upon the removal of the signal peptide region, the sequences were used for the prediction of membrane-spanning regions using TMHMM server v.2.0 (https://www.cbs.dtu.dk/services/TMHMM/)^44^ to eliminate transmembrane proteins. Subsequently, cationic amphipathic helix prediction was performed using HeliQuest (https://heliquest.ipmc.cnrs.fr/)^45^. HeliQuest program slides an 18-residue window along the protein sequence and calculates hydrophobicity, hydrophobic moment, and net charge for each generated segment. Appropriate screening parameters were determined by analyses of cecropin-A (GenBank: AAA29185), moricin (BAB13508), Ci-PAP-A (ABR45664), Ci-MAM-A (ACA97856), dermaseptin SI (CAD92232), and PGLa (CAA25963): hydrophobicity between 0 and 0.6, hydrophobic moment between 0.1 and 1.0, and net charge between 3 and 10. Sequence statistics used in HeliQuest screening were as follows: minimal number of polar residues, minimal number of uncharged residues (serine, threonine, asparagine, glutamine, and histidine), minimal number of glycine residues, and maximal number of charged residues were 6, 1, 0, and 12, respectively, and cysteine was excluded. For identification of an anionic helical region in the LCAMP precursor, parameters were limited: hydrophobicity between − 0.7 and 0.4, hydrophobic moment between 0 and 0.5, and net charge between − 10 and − 3. Secondary structures of putative cationic amphipathic segments were further confirmed by PROTEUS (https://www.proteus2.ca/proteus/)^46^, and their aggregation propensities were calculated using AGRRESCAN (https://bioinf.uab.es/aggrescan/)^47^. Finally, subcellular localization was predicted using DeepLoc-1.0 (https://www.cbs.dtu.dk/services/DeepLoc/) to confirm the secretion of LCAMP candidates^48^.

### Sequence analysis

Peptide sequences were aligned using ClustalW. The prediction of NF-κB binding sites upstream of the initiating methionine codon of each gene (less than 2,000 base pairs) was carried out using Match-1.0 public (https://www.gene-regulation.com/pub/programs.html)^49^. The values of the cut-off for core and matrix similarities were 0.75 and 0.8, respectively.

### Antimicrobial activities of the peptides

Peptides used in this study were chemically synthesized using the Fmoc method and obtained from Eurofins Genomics (Tokyo, Japan). The sequences of synthetic peptides were as follows:

KH.C1.365, RRRRRIAARIGSGVAQTGGELI;

KH.C1.640, WWLSRRRSSLFYWR;

KH.C7.94, SRRRFDFSRRRIYVARRRSLAFAHRRR;

KH.L63.9, FRRRRRRRRHWHHHHHYHYHHHRRRRRRRRRW;

KH.S1531.4, TINKKFRWHGKRKWWLRFVKQ;

KH.S908.1, KGGKFLNFLKKAAKVGAKVGMAALG;

KH.S921.1, KLKTWPKNYWRKVWSKKNWRKFVKKFKHW;

KH.S930.2, AERLMRKAAKDHWSNKMAKDVIWWEQ;

KH.S1007.2, FGRRRRVPGRRRRWWNERAMNEI.

Antimicrobial activities of the peptides were measured as described previously^50^. Synthetic peptides were added to the microbial suspension in the individual wells of a microplate at final concentrations of 3.125, 6.25, 12.5, and 25 μM. The relative activities (RA) were calculated by the equation RA = ((A_PC_ − A_NC_)  − A_SA_)/(A_PC_ − A_NC_), where A_PC_, A_NC_, and A_SA_ represent the absorbance of positive control, negative control, and samples, respectively. The bacteria and yeast strains used were *Escherichia coli* (NBRC14237), *Staphylococcus aureus* (NBRC12732), *Pseudomonas aeruginosa* (NBRC12582), and *Saccharomyces cerevisiae* (NBRC10217), which were purchased from Biological Resource Center, National Institute of Technology and Evaluation (NBRC, Tokyo, Japan). The interaction between *Ciona* LCAMPs and membrane interface was predicted using AmphipaSeek server (https://npsa-pbil.ibcp.fr/cgi-bin/npsa_automat.pl?page=/NPSA/npsa_amphipaseek.html)^28^.

### Hemocyte collection and stimulation

*Ciona* adults were provided by the Maizuru Fisheries Research Station of Kyoto University and by the Misaki Marine Biological Station of the University of Tokyo through the National BioResource Project of MEXT, Japan.

The animals were bled by removal of the tunic and puncture of the heart, and washed with sterile Ca^2+^- and Mg^2+^-free artificial seawater (CMF-ASW; 463 mM NaCl, 11 mM KCl, 25.5 mM Na_2_SO_4_, 2.15 mM NaHCO_3_, 5 mM HEPES (pH 8.0), 5 mM EDTA). The coelomic fluid (hemolymph) and wash solution were collected and filtered through a nylon net. After centrifugation at 1,000×*g* for 30 s, the hemocyte pellet was washed with CMF-ASW and resuspended in CMF-ASW. Coelomic fluid was further centrifuged and the resulting supernatant (hemolymph) was used for the cultivation of hemocytes.

Hemocytes were plated in a 24-well plate and cultured at 16 °C in filtered seawater containing 20% hemolymph in the presence of penicillin (final concentration of 50 units/mL) and streptomycin (final concentration of 50 μg/mL). After incubation for 36 h, the cells were cultured in 20% hemolymph/seawater without antibiotics and then stimulated with 0.1 mg/mL LPS from *E.coli* (Sigma-Aldrich, St. Louis, MO, USA) for 1 h at 16 °C.

### Reverse transcription-polymerase chain reaction (RT-PCR) analysis

Total RNA was extracted from uninduced and LPS-induced cells and was reverse transcribed using PrimeScript RT reagent Kit (TAKARA Bio Inc., Shiga, Japan) with oligo dT primer. The primers used in this study are mentioned in Supplementary Table S2. PCR cycles were as follows: initial denaturation at 94 °C for 2 min; 35 or 40 cycles at 94 °C for 30 s, 48 °C for 30 s, and 68 °C for 30 s. EF1α (elongation factor 1α) and PPIA (peptidylprolyl isomerase A) were used as internal control for RT-PCR analyses. The PCR products were separated by electrophoresis on 2% agarose gel and stained with ethidium bromide.

## Supplementary information

Supplementary file1 (PDF 133 kb)

## Abbreviations

LCAMP: Linear cationic α-helical antimicrobial peptide
MIC: Minimum inhibitory concentration
LPS: Lipopolysaccharide
EF1α: Elongation factor 1α
PPIA: Peptidylprolyl isomerase A
