# Supplementary material for: In silico identification and functional validation of linear cationic α-helical antimicrobial peptides in the ascidian Ciona intestinalis
Source: Sci Rep. 2020 Jul 28;10:12619. doi: 10.1038/s41598-020-69485-y (PMC7387483; doi:10.1038/s41598-020-69485-y)
Supplement: Supplementary file 1 — Supplementary file1 (PDF 133 kb) [file 41598_2020_69485_MOESM1_ESM.pdf]

Supplementary information

In silico **identification and functional validation of linear cationic α-helical antimicrobial peptides in the ascidian *Ciona intestinalis***

Yukio Ohtsuka and Hidetoshi Inagaki

Biomedical Research Institute, National Institute of Advanced Industrial Science and Technology (AIST), 1-1-1 Higashi, Tsukuba, Ibaraki 305-8566, Japan

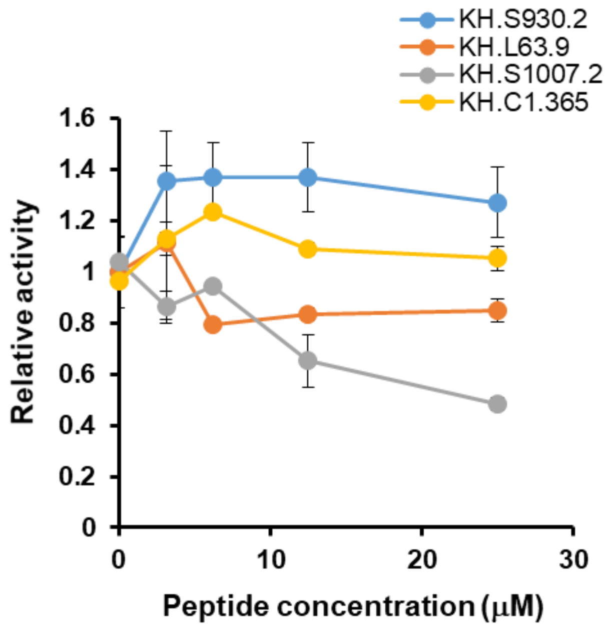

Supplementary Figure S1. Antimicrobial activity of cationic amphipathic peptides with lower aggregation propensity.

Antimicrobial activity of KH.C1.365, KH.L63.9, KH.S1007.2, and KH.S930.2 peptides with Na^4vSS value less than -30 against *E. coli* was tested at various concentrations. Error bars show the standard deviation ($n = 3$) from a single experiment.

Supplementary Table S1. List of the predicted LCAMPs from *X. tropicalis* genome database.

Gene ID	Cationic amphipathic helix sequence	Putative protein product
112419089	MAKYYSALRHYINLITRQRYGKRSSPET	neuropeptide Y
113197744	MAKYLTALRHYINLVTRQRYGKRDSPA	peptide YY
284520880	VRGLLSNVAGLLKQFAKGGVNAVLNPKR	preproprotein pGQ preproprotein / xpf-St7*
289470300	NERVARNLLGSLLKTGLKVGSNLLGRREANDRRF	XT-6 like precursor (xt6l) / cpf-st7*
301623560	EAMTKTRNWFTDLYKKIKEKTTGS	hypothetical protein LOC100489130
301624773	IEREVRGFMSKVANFAKKFAKGGVNAIMNQKREAM FNSAHSKRFIGALLRPAL	prolevitide-like / xpf-St8*
301624775	REVRGVWSTILGGLKKFAKGGVNAVLNPKR	xenopsin-like
301624779	RFTRGWASSIGSILGKFAKGGAQAFLQPKR	prolevitide-like / xpf-St4*
301624781	ARGLKEVAHSAKKFAKGFISGLTGSKREAMLRS	magainins-like / magainin-st1*
301624785	NERVARGVFGLLAKAALKGASKLIPHL	hypothetical protein LOC100489440 / cpf-st5*
301625556	RSENRNLFLSALQSYFRRRGIAI	hypothetical protein LOC100498126

* nomenclature of LCAMPs by Roelants K. *et al.*.

Supplemental Table S2. Primer sequences and PCR cycle numbers

Gene ID	Gene name	Forward primer	Reverse primer	cycle number
KH.C1.100	*Ci-MAM-A*	GCTCTTCTTCTCTTGGTGTC	CACTTTGTTTCGTTCAATGACAAG	40
KH.C1.453	*Ci-PAP-A*	GATCCCTATGTCAGAACC	CGTAGAGCTGGTGTTTGC	35
KH.C1.640		CTCATCGTTCTTCTCATCGC	TCAAAAGCGAGAAAGATTAGTT	35
KH.C14.152	*Ci-META4*	GATTCCGTGGGCTTGTTC	GGTGTAATCTCGAAATGTTG	35
KH.C14.235	*Ci-META4*	GCCACCCTTCCTTCAGAG	CGAGAAAACGTGAGGTGTAA	35
KH.C7.94		GAACATGAAACGAAATGCTGT	TCAAGTTTACATTTTAAAAGTCT	35
KH.S1531.4		ACAAATATTTAGCTATGGACAGA	GTTTGTTGACAGGATG	40
KH.S908.1		ATATCTCTTACTTCATCTGAAAC	TTGGTGATTGTGTTTTATAT	35
KH.S921.1		ATGAACAAGTCAGCACTTCT	TTTAAATCGTACGATCTTTATTG	35
KH.C14.52	*Ci-EF1a*	GGCACTGGTGGTAAGAGCAT	GGAGCCAACACCCTCAACTT	35
KH.C9.410	*Ci-PPIA*	TTTCGCTGTCCGTGACATGA	AAAGTGTTTTCGGTGGGGGT	35

Supplementary Reference

Roelants, K. et al. Origin and functional diversification of an amphibian defense peptide arsenal. *PLoS Genet* **9**, e1003662 (2013).

Full-length gel images which are source data for Figure 3.

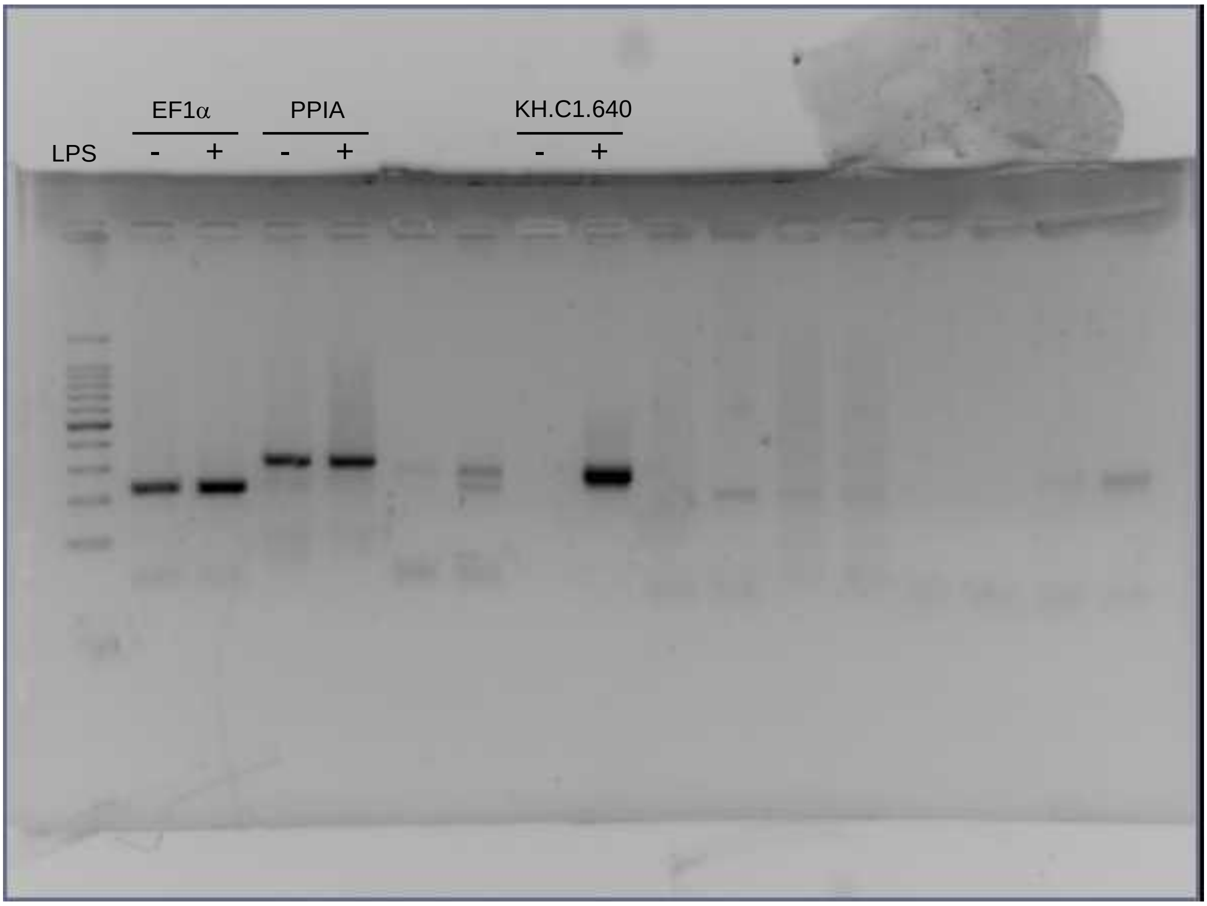

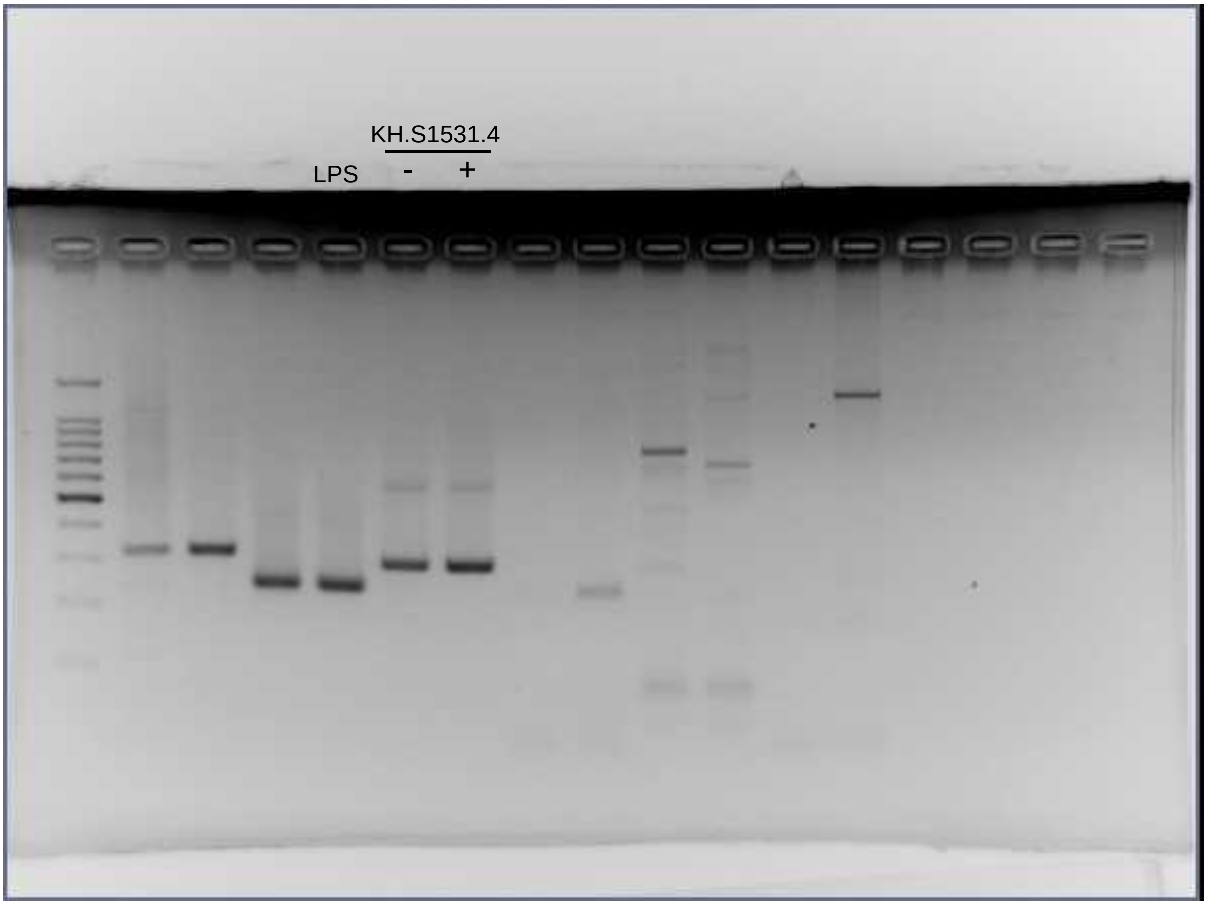

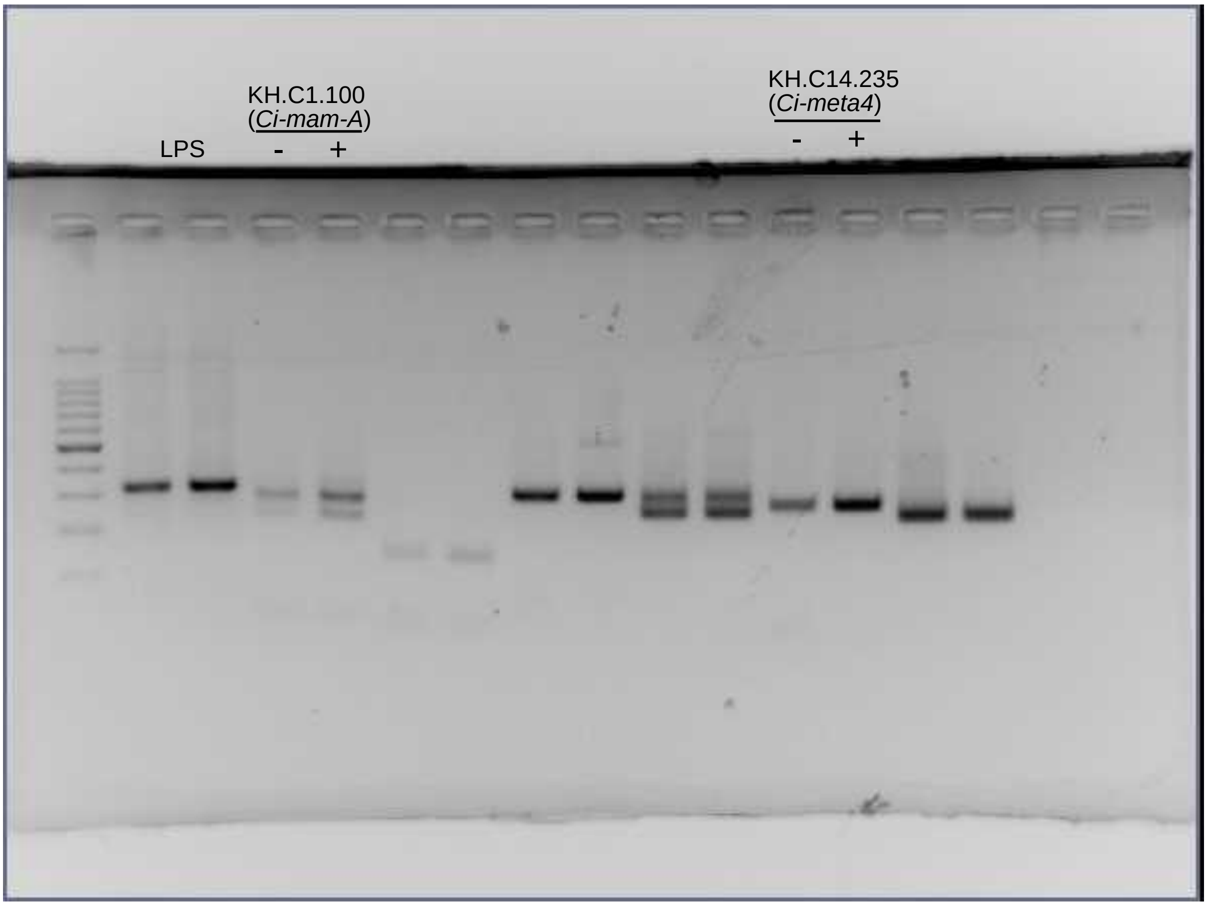
KH.C1.100
(Ci-mam-A)
LPS
- +
KH.C14.235
(Ci-meta4)
- +

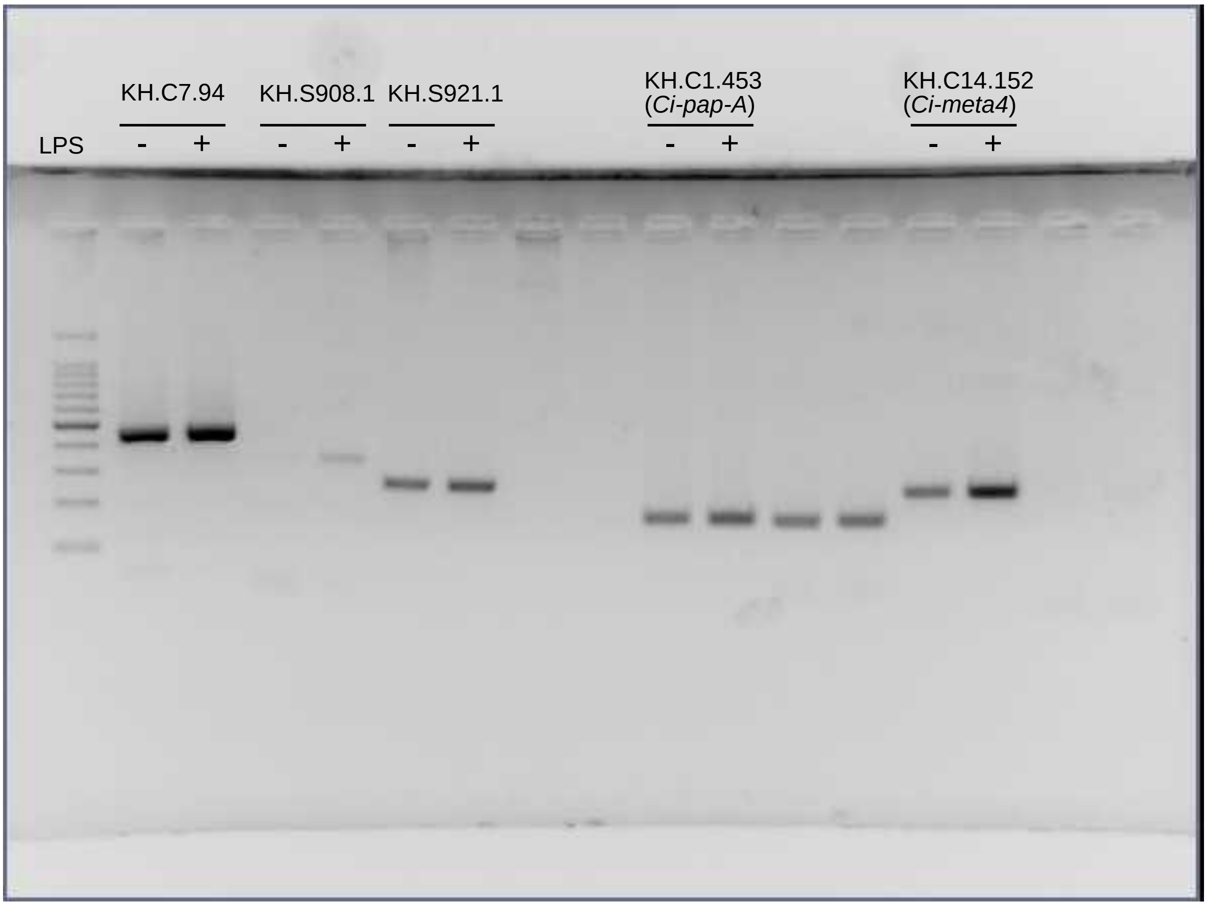
KH.C7.94
KH.S908.1
KH.S921.1
KH.C1.453
(Ci-pap-A)
KH.C14.152
(Ci-meta4)
LPS
- + - + - + - + - +
